# Sarcopenia: Molecular regulatory network for loss of muscle mass and function

**DOI:** 10.3389/fnut.2023.1037200

**Published:** 2023-02-02

**Authors:** Jiaxiang Wu, Ping’an Ding, Haotian Wu, Peigang Yang, Honghai Guo, Yuan Tian, Lingjiao Meng, Qun Zhao

**Affiliations:** ^1^The Third Department of Surgery, The Fourth Hospital of Hebei Medical University, Shijiazhuang, China; ^2^Hebei Key Laboratory of Precision Diagnosis and Comprehensive Treatment of Gastric Cancer, Shijiazhuang, China; ^3^Research Center of the Fourth Hospital of Hebei Medical University, Shijiazhuang, China

**Keywords:** sarcopenia, molecular mechanism, neuromuscular junction lesion, imbalance of protein synthesis and breakdown, satellite cells dysfunction

## Abstract

Skeletal muscle is the foundation of human function and plays a key role in producing exercise, bone protection, and energy metabolism. Sarcopenia is a systemic disease, which is characterized by degenerative changes in skeletal muscle mass, strength, and function. Therefore, sarcopenia often causes weakness, prolonged hospitalization, falls and other adverse consequences that reduce the quality of life, and even lead to death. In recent years, sarcopenia has become the focus of in-depth research. Researchers have suggested some molecular mechanisms for sarcopenia according to different muscle physiology. These mechanisms cover neuromuscular junction lesion, imbalance of protein synthesis and breakdown, satellite cells dysfunction, etc. We summarize the latest research progress on the molecular mechanism of sarcopenia in this review in order to provide new ideas for future researchers to find valuable therapeutic targets and develop relevant prevention strategies.

## 1. Introduction

Sarcopenia was described as a syndrome by the European Working Group on Sarcopenia in Older People (EWGSOP) in 2010, which was characterized by degenerative changes in skeletal muscle mass and function throughout the body (1). However, sarcopenia is recognized as a muscle disease in the EWGSOP2 Consensus updated in 2019 (2). The guidelines (1, 2) state that decreased muscle strength as an important sign of sarcopenia should be examined first when a patient is suspected of sarcopenia. A patient is diagnosed with sarcopenia when both muscle strength and muscle mass are reduced, while a loss of muscle strength alone is pre-sarcopenia. In addition, muscle performance is an important indicator of the severity of disease.

Sarcopenia and cachexia are generally difficult to distinguish clinically in elderly cancer patients due to the common features of muscle atrophy (3). However, Berardi et al. (4) found that unlike cachexia, which features gradual loss of lean mass and adipose tissue in the absence of substantial muscle protein loss, sarcopenia is usually accompanied by chronic protein loss. Furthermore, systemic inflammation plays an important role in the occurrence and development of cachexia, which can be used to identify cachexia (5). In contrast, sarcopenia is often associated with age-related chronic low-grade inflammatory profile, and can occur even in patients without inflammation (4, 6). Therefore, the in-depth exploration of the mechanism of the occurrence and development of sarcopenia can contribute to the clinical differential diagnosis.

The quality and function of skeletal muscle are affected by a variety of molecular pathways such as aging, inflammation, malnutrition, oxidative stress, and mitochondrial dysfunction, thus accelerating the occurrence and development of sarcopenia (1, 2). However, the study of the molecular mechanism of sarcopenia is still limited due to the complexity and interrelation of these molecular pathways. It is now widely identified that the increase of age is a central element affecting the development and progression of physiological sarcopenia (7, 8). Age is not only a risk factor for diseases such as diabetes, cardiovascular disease, and cancer, but also a risk factor for sarcopenia (9–12). The main mechanism of skeletal muscle aging is muscle fiber atrophy and loss from the perspective of tissue level, while the muscle fiber atrophy is mainly caused by the imbalance between muscle protein synthesis and breakdown (13). Moreover, sarcopenia has recently been recognized as one of the criteria for the diagnosis of malnutrition by the Global Leadership Initiative on Malnutrition (GLIM) (14). Schneider et al. (15) have shown that sarcopenia can be used as a substitute for malnutrition and debilitating symptoms in patients, which further confirmed that sarcopenia was closely related to malnutrition.

Sarcopenia increases the mortality and disability rate of the elderly, affects the prognosis of patients, and increases the economic burden of patients (16). Herein, we discuss the fundamental molecular mechanisms of the occurrence and development of sarcopenia. Based on these studies, we are expecting to clarify its internal changes, develop prevention strategies, and identify molecular targets for future therapeutic interventions.

## 2. Neuromuscular junction lesion

Neuromuscular junction disease can lead to decreased muscle function through neuronal degeneration and muscle fiber denervation, which is one of the most important causes of sarcopenia (17). The neuromuscular junction (NMJ) is composed of three main components: the presynaptic motor nerve terminals; the synaptic cleft; and the endplate (18).

Agrin is released by motor neurons (19). Action potentials can be correctly transmitted from nerve endings to muscle through the agrin-muscle specific kinase (MuSk)-lipoprotein receptor-related protein 4 (Lrp4) signaling pathway, which is essential for maintaining normal neuromuscular function (20). Meanwhile, the agrin-MuSk-lrp4 signaling pathway mediates the aggregation of acetylcholine (ACh) receptors on the postsynaptic membrane (21). A growing number of research results have demonstrated this signaling pathway is dysfunction with the age increase, leading to changes in NMJ structure and function (18, 22, 23).

Deschenes et al. (24) proposed that acetylcholine release increased in aged muscles leading to a faster rate of neuromuscular fatigue. It has been suggested that decreasing ACh may be an effective remedy to treat structural changes in NMJ that can lead to sarcopenia (25). The same result was obtained in another study, which found that moderately reduced ACh levels could increase NMJ area, myofiber cross-sectional area, and satellite cells number in mice (26). Interestingly, Castets et al. (27) reported that the mechanistic target of rapamycin (mTOR) signal transduction also has a positive effect in the structural formation and functional maintenance of NMJ. This evidence may suggest that NMJ functional changes in sarcopenia are related to decreased protein synthesis. However, the molecular mechanism is still unclear and needs to further study.

## 3. Satellite cells dysfunction

As a key pathway for skeletal muscle satellite cell differentiation, wnt signaling pathway can stimulate skeletal muscle development and regeneration during the embryonic period, which is an extremely coordinated process (28). Studies targeting the typical wnt/β-catenin pathway have shown that the number of proliferative satellite cells increases with activation of the pathway and that the pathway can inhibit skeletal muscle atrophy under the regulation of the zinc finger transcription factor Kruppel-like factor 5 (KLF5) (29, 30). However, several researchers have recently demonstrated that hyperactive wnt/β-catenin signaling pathway can also lead to damage of skeletal muscle stem cells and inhibition of muscle regeneration (31, 32). These evidences suggest that balancing the expression of the canonical wnt pathway is an essential prerequisite for muscle regeneration.

Growing evidence indicated that the Type II muscle fiber atrophy leads to muscle mass loss in the sarcopenia, and the loss of satellite cells may be a significant cause of type II myofiber atrophy (33–35). Furthermore, Laura et al. found that the function and number of skeletal muscle stem cells decreased with age (36). A recent study suggests that the p53-p21CIP1 and P16INK4a-retinoblastoma (Rb) pathways may be the major signaling pathways for the activation of these stressors (37). Downstream studies on the two pathways have shown that cyclin-dependent kinase (CDK) 2 and CDK4/6 may act as downstream inhibitory molecules to promote over-phosphorylation of Rb protein, which can ultimately terminate cell division and thus enter the senescence state (37). The upstream signals of both pathways have been widely studied in satellite cells. A recent study demonstrated that loss of zinc-finger transcription factor Slug, which was a direct transcriptional repressor of p16^InK4a^, could cause satellite cells to enter complete senescence under damage-induced stress (38). Furthermore, Myers et al. (39) reported that sirtuin 1 (SIRT1) and p53 have a synergistic effect in protecting skeletal muscle against fatigue damage, and type I fibers can also be protected from atrophy damage by this effect. Accordingly, SIRT1 may selectively inhibit the mechanism of p53 fiber damage, whereas further study needs to be done to confirm this conclusion. In contrast to the above views, Fry et al. (40) found that both mice injected with tamoxifen to clear satellite cells and control mice developed sarcopenia, and muscle fiber atrophy and grip strength were not changed by clearing satellite cells. Meanwhile, an increase in fibrosis was observed in skeletal muscle deprived of satellite cells. Therefore, the role of decreased regenerative capacity caused by senescence of satellite cells in senile sarcopenia needs further study.

## 4. Decreased protein synthesis

**FIGURE 1:**
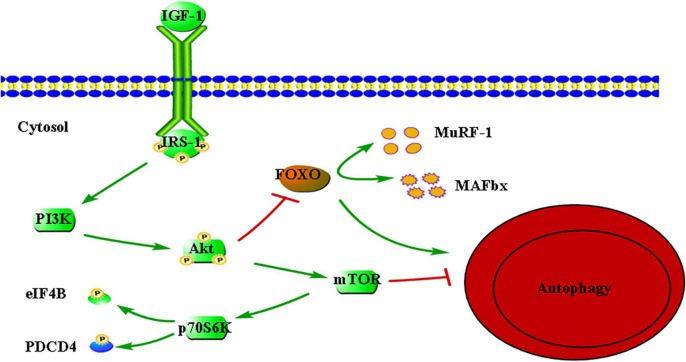
Insulin-like growth factor 1 (IGF-1)-mediated signaling pathways relevant to protein synthesis. IGF-1, insulin-like growth factor 1; IRS-1, insulin receptor substrate 1; PI3K, phosphoInositide-3 kinase; Akt, protein kinase B; mTOR, mechanistic target of rapamycin; p70S6K, 70-kDa ribosomal protein S6 kinase; eIF4B, eukaryotic initiation factor 4B; PDCD4, programmed cell death 4; FOXO, O-type fork head box; MuRF-1, muscle RING-finger protein-1; MAFbx, muscle atrophy F-Box. Arrow indicates activation of expression; lines with a perpendicular line at the end indicate inhibition of expression.

Currently, a large amount of studies have been done to explore the molecular mechanism of insulin-like growth factor 1 (IGF-1) participating in protein synthesis (41–45). The IGF-1 is an essential factor of metabolic molecular signaling, which is involved in a variety of skeletal muscle anabolic pathways. IGF-1 first binds to and *trans* phosphorylates the transmembrane tyrosine kinase receptor (IGFR) to form the docking site of insulin receptor substrate 1 (IRS-1). IGFR subsequently binds IRS-1 to activate the phosphoInositide-3 kinase (PI3K)-protein kinase B (Akt) pathway. Akt can activate mammalian target of rapamycin (mTOR) through phosphorylation and inactivates tuberous sclerosis complex-2. Subsequently, the 70 kDa ribosomal protein S6 kinase (P70S6K) is phosphorylated and activated by mTOR. Finally, eukaryotic initiation factor 4B (eIF4B) and programmed cell death 4 (PDCD4), which are two proteins that modulate cap-dependent mRNA translation are phosphorylated by activated p70S6K subsequently, resulting in increased mRNA translation either directly or indirectly. In addition, the IGF-1-PI3K-Akt pathway can also phosphorylate and inactivate O-type fork head box (FOXO) transcription factors to prevent protein degradation (Figure 1). FoxO1, FoxO3, and FoxO4 can promote the activation of protein breakdown mechanism with increasing the transcription of muscle ring finger protein-1 (MuRF-1) and amyotrophic F-box (MAFbx) genes, which are three isoforms expressed in skeletal muscle. It should be noted that Akt requires the simultaneous integration of FOXO1 and mTORC1 pathways to maintain oxidative metabolism and protein synthesis, which are not parallel (46).

Ascenzi et al. (47) found that molecular markers related to the autophagy pathway were significantly up-regulated in muscles of aged IGF-1Ea and IGF-1Eb mice. In addition, the two isoforms could also promote the expression of mitochondrial fusion proteins and mitochondrial fission process protein 1, which is a downstream target of a factor (PGC1-α) with antioxidant stress, to promote mitochondrial fusion and fission. The above evidence suggest that IGF-1 isoforms can counteracts sarcopenia by activating a series of molecular pathways that maintain skeletal muscle anabolism and regeneration. However, they found that only IGF-1Ea could promote skeletal muscle enlargement when both isoforms were overexpressed. This result implies that the two subtypes may mediate different molecular mechanisms in skeletal muscle, which needs further research to confirm.

In recent years, decreased levels of IGF-1 have been found to be associated with sarcopenia in various disease states (48–50). Xu et al. (51) found that patients with sarcopenia were older (*P* < 0.001) and had significantly lower IGF-1 levels (*P* < 0.01) through measuring the hormone levels of patients, suggesting that aging may affect IGF-1 levels. This is the same result as the study by Naro et al. (52). However, Naro et al. found the expression of genes in muscles of the elderly that could cause muscle fiber atrophy were also decreased significantly. The above evidences suggest that reduced protein anabolic metabolism is closely associated with sarcopenia, while it seems that it can play a compensatory role by downregulating atrophic factors in the elderly, thereby partially restoring protein stability.

## 5. Ubiquitin proteasome

**FIGURE 2:**
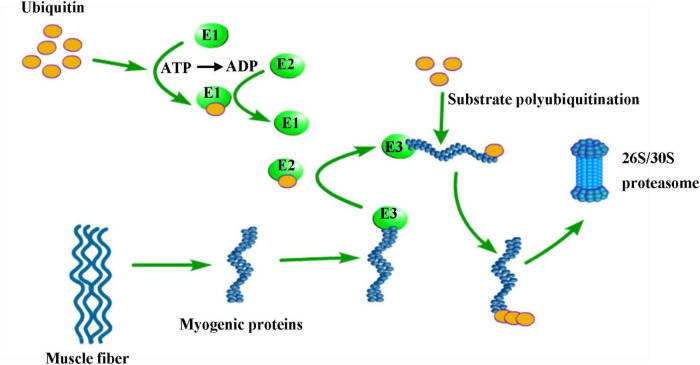
Degradation of muscle fiber proteins by the ubiquitin-proteasomal system. E1 (ubiquitin activating enzyme), E2 (ubiquitin binding enzyme) and E3 (ubiquitin ligase) gradually bind to ubiquitin resulting in E3 activation, which is accompanied by adenosine triphosphate (ATP) consumption. Activated E3 ubiquitin ligases can recognize myogenic proteins. Several molecules of ubiquitin are bound to the substrates in a K48 poly-ubiquitin chain. This poly-ubiquitin chain then binds to the 26S/30S proteasome, where the substrate protein is degraded to short peptides and amino acids.

Ubiquitin proteasomes (UPS) and autophagic lysosomes are two proteolytic systems that control protein degradation in skeletal muscle (53). Proteins are ubiquitinated by ubiquitin activator enzyme (E1), ubiquitin-binding enzyme (E2), and ubiquitin ligase (E3), and then degraded into short peptides and amino acids in the 26S/30s proteasome (42) (Figure 2). E3 is a key enzyme in this pathway, which can identification specific target protein sequences and precisely regulate the degradation rate of the target protein. Atrogin-1 (MAFbx) and MURF-1 are two important E3 ubiquitin ligases, which are regarded as markers of skeletal muscle atrophy. Researchers have observed their expression in a variety of diseases that can result in skeletal muscle atrophy (54).

Recently, there are increased evidences have demonstrated that the level of oxidative stress and chronic inflammation is an essential factor in inducing sarcopenia in the elderly (55, 56). Some studies have shown that the p38 mitogen-activated protein kinase (MAPK) and nuclear factor-Kappa B (NF-KB) paths are two basic pathways for UPS activation, which can be activated and regulated by oxidative stress and inflammatory cytokines, such as tumor necrosis factor- α (TNF- α) And interleukin-1 (IL-1) (57). In another study, excessive reactive oxygen species (ROS) and inflammatory factors can activate UPS and autophagy processes through phosphorylation signal transducers and transcriptional activators 3 (58).

Wu et al. (59) found that the expression of MAFbx or MuRF-1 gene in normal young muscle was similar to the elderly. However, Bodine and Baehr (60) found that proteasome and other components of UPS increased in muscle with researching sarcopenia rats. The study of Zhang et al. (61) also obtained the same result, which found that MuRF-1 expression and protein ubiquitination were higher in patients with sarcopenia than in non-sarcopenia patients, while atrogin-1 expression was not significantly different. Meanwhile, Sharma et al. (62) found that T. cordifolia water extract could reduce oxidative stress and inflammation by inhibiting MURF-1 and calpain, which can reduce proteolysis. The above evidence indicates that UPS activated mainly in patients with severe muscle atrophy, and MuRF-1 plays a major role in skeletal muscle degradation. UPS has a promising future as a therapeutic target for sarcopenia.

The proteolysis effect of UPS in inducing muscle mass loss has been widely confirmed. However, studies have also shown that UPS plays an important role in maintaining muscle mass. Three isogenies of MuRF, MuRF-1, MURF-2, and MuRF-3, are expressed in myocardial and skeletal muscle as the key factor E3 of UPS (63). Perera et al. (64) showed myogenic differentiation and delayed myotube formation after MURF-2 knockout in mice. Lodka et al. (65) found that the muscle strength of soleus and extensor digitalis longus in MuRF-2 and MuRF-3 double-knocked mice was weakened, and the fiber type changed to type I. Moriscot et al. (66) found significant loss of sodium type ii fibers in mice with MuRF1 and MuRF2 knocked out and showed that the maintenance effects of MuRF-1 and MuRF-2 on type ii fibers were closely related to myozenin-1. The above evidence suggests that the MuRF family plays an important role in the maintenance of early differentiation, structure, and function of skeletal muscle. It remains to be seen whether the UPS-related gene knockout alone can benefit patients with sarcopenia.

## 6. Autophagy

In recent years, autophagy, which is a protein degradation mechanism other than UPS, has been extensively studied in relation to sarcopenia (67, 68). Pigna et al. (69) have demonstrated that maintaining a balance of autophagy production and clearance guarantees adequate autophagy fluxes in muscles, and this balance is disrupted in colon cancer mice, leading to incorrect autophagy fluxes and promoting muscle consumption. This result suggests that the disruption of autophagy balance may induce sarcopenia.

Aging may be an important cause of autophagy defects among the factors that break the balance. A recent study has shown that the clearance capacity of autophagy lysosome system (ALS) decreases with aging, resulting in insufficient clearance of accumulated wastes by cells (70). Moreover, studies have found that autophagy defects in aged stem cells could reduce the regenerative ability of satellite cells in sarcopenia, and the activation of autophagy clears senescent cells in time to promote muscle regeneration (71, 72).

Excessive autophagy caused by disease states such as cancer and malnutrition may be another important cause of autophagy imbalance. Zhang et al. (61) confirmed that the protein expressions of Beclin-1 (the autophagy key regulatory protein complex) and LC3B (LC3-II and LC3-I) were up-regulated in sarcopenia patients with or without gastric cancer, suggesting ALS is activated. Furthermore, it has demonstrated that malnutrition can inhibit the PI3K-Akt signaling pathway in mouse muscles, which can subsequently induce autophagy through reducing mTOR and activating FOXO3, resulting in excessive autophagy (53).

In addition to affecting muscle mass, muscle function can also be impaired by the breakdown of autophagy balance due to the dysfunction of autophagy in the process of mitochondrial clearance (73). Mitophagy selectively degrades mitochondria through the Pten-induced putative kinase 1 (PINK1)/Parkin pathway and BCL2 adenovirus E1B 19-KDA-Interacting protein 3 (BNIP3)/BNIp3-like (BNIP3L, also known as NIX) pathway, thereby eliminating dysfunction and redundant mitochondria to maintain energy homeostasis. The BNIP3/NIX pathway directly interacts with LC3-II through the connexin p62 (74–76). Urine-varela et al. (74) found that the molecular expression of mitochondrial autophagy signaling pathway decreased in the process of aging, leading to mitochondrial dysfunction and ros induced biomolecular damage increased. Meanwhile, Davuluri et al. (76) demonstrated that mitochondrial phagocytosis increases with age, resulting in a decrease in mitochondrial content in aging muscle. According to the above evidence, maintaining equilibrium and efficient autophagy is crucial for preserving muscle homeostasis.

## 7. Nutrition

Endoplasmic unfolded protein response (UPR) is the general name of the signaling pathway induced by endoplasmic reticulum (ER) stress (77). Hulmi et al. (78) found that UPR/ER pathway indicators such as activated transcription factor-6 (ATF6), inositol requirement protein (IRE) 1α, and protein kinase R (PKR) -like endoplasmic reticulum kinase (PERK) protein were increased and muscle strength was decreased in a mouse model of malnutrition. Meanwhile, levels of multiple UPR markers in skeletal muscle of fasting mice were found to be decreased with the loss of TNF receptor-associated factor 6(TRAF6), an E3 ubiquitin ligase (79). These studies suggest that UPR may be involved in skeletal muscle atrophy caused by nutritional deficiency. However, the factors that activate UPR in malnutrition and the specific mechanisms of the various branches of UPR in skeletal muscle atrophy remain unclear and require further investigation.

Amino acids are primarily stored in skeletal muscle. Previous studies have shown that basic amino acid metabolism may not be affected during aging, while the ability to stimulate anabolic responses is reduced in older adults (80). Essential amino acids (EAA) and branched-chain amino acids can stimulate protein synthesis in the elderly. Leucine can directly initiate mRNA translation and play a key role in regulating human and rat muscle protein synthesis (81). However, the effect appears to diminish in older adults. Similar to this argument, Drummond et al. (82) found that the muscle protein synthesis (MPS) response of the elderly was slower than that of the young after resistance exercise and EAA intake, which may be related to the non-response of ERK1/2 signal and AMPK activation in the aging muscles. This anabolic resistance is closely associated with loss of muscle mass, strength, and function (83). However, further research is needed to elucidate the upstream drivers of this anabolic resistance. In addition, Brook et al. (84) have shown that muscles need to provide other tissues with amino acids and other substrates due to the huge energy requirements of patients with nutrient deficiencies, which are mainly manifested as accelerated catabolism and reduced synthesis of muscle protein, while protein synthesis of liver increases. This means that skeletal muscle tissue may be continuously depleted and reduced in some disease states due to disruption of amino acid balance in the body.

Combining a nutritional strategy of amino acid supplementation with exercise may be the best treatment strategy for people with age-related sarcopenia (85). Leucine supplementation may aid in muscle protein synthesis in the elderly due to its important role in protein synthesis. Xu et al. (86) have shown that leucine supplementation can ameliorate the sluggish muscle response to amino acid intake in older adults and contribute to increased protein synthesis after meals. Furthermore, a recent study suggests that higher intake of branched chain amino acids may help prevent sarcopenia in older adults (87). Studies have shown that physical activity can regulate oxidative stress and satellite cell activity, increase muscle’s ability to adapt to oxidative stress, and promote the proliferation of satellite cells, thus delaying the development of sarcopenia (88). In addition, resistance training can increase the cross-sectional area of type II muscle fibers and improve aging muscle outcomes and function (29).

Caloric restriction (CR) may be a measure to prevent the adverse effects of aging for the elderly. CR and maintenance of adequate micronutrient supply have been found to play a positive role in extending life span and alleviating aging-induced sarcopenia in mouse models (89, 90). However, caloric restriction should probably be used to limit calorie intake while providing adequate protein since malnutrition is common in many elderly patients with sarcopenia, and increased amino acid intake may delay sarcopenia (43).

## 8. Inflammation and metabolism

Studies have shown that age-related muscle loss is often associated with chronic low-grade inflammation (6). This effect is often associated with inflammatory factors that can cause imbalances in muscle protein synthesis and breakdown, which can lead to muscle atrophy (91, 92). In multiple studies of the relationship between inflammation and sarcopenia, levels of the inflammatory markers C-reactive protein, TNF-α, and IL-6 have been negatively correlated with muscle strength and mass (93, 94).

Age-related sarcopenia may be accompanied by an increase in fat content along with a decline in muscle strength and mass (95). In response to aging and chronic inflammation, the increased fat content in the body is redistributed, resulting in increased visceral fat content and infiltration of skeletal muscle fat (96). The increase of fat infiltration in and between myocytes can induce mitochondrial dysfunction, resulting in the increase of ROS produced by lipid oxidation, and promote a series of adverse reactions such as lipid toxicity, insulin resistance (IR), and local inflammation (97). Under the combined action of ROS and local inflammation, oxidative stress is enhanced and IGF-1-PI3K-mTOR pathway is inhibited, leading to reduced protein synthesis while mitochondrial function is further damaged, forming a vicious cycle (97). Meanwhile, pro-inflammatory factors released by local inflammation can induce infiltration of macrophages and other immune cells, further release a large number of inflammatory factors, promote the breakdown of muscle protein, expand the range of inflammation, and aggravate the damage of IR and lipotoxicity (98). In addition, lipid toxicity has been shown to inhibit protein synthesis and promote anabolic resistance by inhibiting phosphorylation of Akt and mTOR through ceramides (99).

Studies have shown that IR leads to increased gluconeogenesis, which promotes increased triglyceride production and transport to skeletal muscle and liver, resulting in increased lipid accumulation in skeletal muscle and liver (97, 100). In addition, IR can cause compensatory hyperinsulinemia with increased myostatin production and inhibition of the IGF-1 axis, resulting in increased muscle protein breakdown, and decreased synthesis (97, 100).

## 9. Conclusion

**FIGURE 3:**
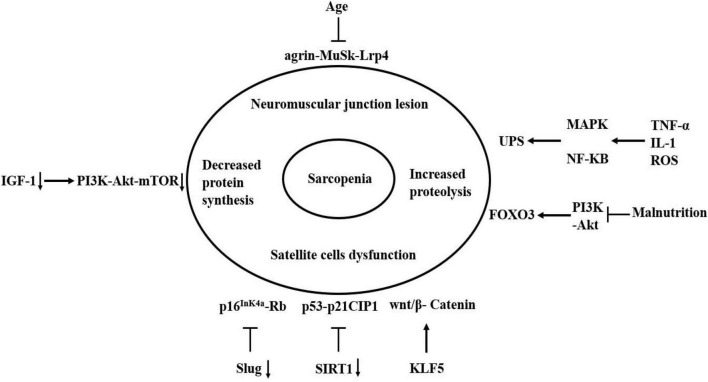
Molecular mechanism of sarcopenia. The agrin-MuSk-Lrp4 signaling pathway may be dysregulated with the age increase, resulting in the neuromuscular junction lesion. Slug and SIRT1 inhibit cell senescence by inhibiting the two signaling pathways of p53-p21CIP1 and p16^InK4a^-Rb. The absence of two factors accelerates the entry of satellite cells into a state of complete senescence under damage-induced stress. The KLF5 can regulate skeletal muscle atrophy through wnt/β-Catenin signaling pathway. The decrease of IGF-1 leads to a decrease in the activation of PI3K-Akt-mTOR signaling pathway, which result in the decrease in protein synthesis. Oxidative stress and inflammatory cytokines can activate and regulate MAPK and NF-KB pathways, which can activate the UPS. Malnutrition can inhibit the PI3K-Akt signaling pathway, which subsequently activates autophagy by activating FOXO3. Arrow indicates activation of expression; lines with a perpendicular line at the end indicate inhibition of expression.

Sarcopenia is a common disease leading to poor prognosis in the elderly, which occurrence is the result of multiple factors. The essence of sarcopenia is a reduction in the cross-sectional area of muscle fibers and the imbalance of protein synthesis and degradation. Currently, the molecular mechanism of sarcopenia has not been fully understood. However, the molecular mechanism of sarcopenia can be distinguished on the basis of etiology due to the in-depth research on the factors affecting the occurrence and development of sarcopenia in recent years (Figure 3).

In conclusion, the mechanism of sarcopenia involves several aspects, including neuromuscular connection damage, satellite cell dysfunction, reduced protein synthesis, increased protein breakdown, and nutrient deficiency. Precisely, aging is considered to be a key factor in sarcopenia. The protein degradation increases with aging, resulting in an imbalance between protein synthesis and breakdown that promotes the loss of muscle mass.

Taking corresponding measures (such as exercise, nutritional supplements, antioxidants, etc.) to intervene in sarcopenia-related molecular mechanism targets is the fundamental direction of future research on the prevention and treatment of sarcopenia. For example, an increasing number of studies indicate that interfering with the IGF-1 pathway can increasing protein synthesis, which may improve sarcopenia (101, 102). Further elucidation of the mechanism of sarcopenia will be helpful in determining effective lifestyle and therapeutic interventions.

## Author contributions

QZ: conception and design, and administrative support. JW, PD, PY, HG, and YT: provision of study materials and patients. PD, PY, YT, and HG: collection and assembly of data. JW and PD: data analysis and interpretation. All authors wrote and approved the final version of manuscript.
